# Useful pharmacodynamic endpoints in children: selection, measurement, and next steps

**DOI:** 10.1038/pr.2018.38

**Published:** 2018-04-18

**Authors:** Lauren E Kelly, Yashwant Sinha, Charlotte I S Barker, Joseph F Standing, Martin Offringa

**Affiliations:** 1 Department of Pediatrics and Child Health, Max Rady College of Medicine, University of Manitoba, Winnipeg, Manitoba, Canada; 2 Therapeutic Goods Administration, Department of Health, Sydney, Australia; 3 Infection, Inflammation and Rheumatology Section, UCL Great Ormond Street Institute of Child Health, University College London, London, UK; 4 Child Health Evaluative Sciences, Research Institute, The Hospital for Sick Children, University of Toronto, Toronto, Ontario, Canada

## Abstract

Pharmacodynamic (PD) endpoints are essential for establishing the benefit-to-risk ratio for therapeutic interventions in children and neonates. This article discusses the selection of an appropriate measure of response, the PD endpoint, which is a critical methodological step in designing pediatric efficacy and safety studies. We provide an overview of existing guidance on the choice of PD endpoints in pediatric clinical research. We identified several considerations relevant to the selection and measurement of PD endpoints in pediatric clinical trials, including the use of biomarkers, modeling, compliance, scoring systems, and validated measurement tools. To be useful, PD endpoints in children need to be clinically relevant, responsive to both treatment and/or disease progression, reproducible, and reliable. In most pediatric disease areas, this requires significant validation efforts. We propose a minimal set of criteria for useful PD endpoint selection and measurement. We conclude that, given the current heterogeneity of pediatric PD endpoint definitions and measurements, both across and within defined disease areas, there is an acute need for internationally agreed, validated, and condition-specific pediatric PD endpoints that consider the needs of all stakeholders, including healthcare providers, policy makers, patients, and families.

Expanding the evidence base for rational drug use in children presents numerous challenges, including a paucity of high-quality research on commonly used medications, complexities in designing and conducting clinical trials (CTs), and a lack of robust data on how developmental changes and disease progression affect drug exposure and response (1). Although pediatricians endeavor to deliver evidence-based pharmacotherapy, high-quality pharmacokinetic (PK) and pharmacodynamic (PD) data to inform patient management are frequently lacking. Without appropriate data on relationships between drug exposure and drug response in children and neonates, healthcare practitioners have insufficient information to definitively maximize the therapeutic benefit when prescribing drugs, while minimizing the toxicity (2).

## Pediatric pharmacodynamic measures: what are they?

PD is broadly defined as “what the drug does to the body” and is often characterized as drug response. In contrast, PK is “what the body does to the drug”. PD endpoints measure a drug’s activity in the body using biomarkers and/or clinical outcomes to quantify efficacy and safety (3). Although various measures of drug response are used by clinicians every day to guide therapy, PD endpoints in CTs are most often parameterized (e.g., turned into a score) so that the treatment effects can be quantitatively compared across studies. For example, a clinical question relating to mild, moderate, and severe symptoms may be transformed to a numeric rating scale from 1 to 10. This concept is discussed further in the section on Outcome-Scoring Systems.

The relationship between drug exposure and PD endpoints has been inadequately studied in children (4, 5). Importantly, developmental (ontogenetic) changes can affect how a drug is absorbed, distributed, and cleared from the body. This developmentally dependent variability in drug exposure affects both the desired pharmacological response and the risk of adverse effects (6, 7). As “pediatric” patients represent extremely diverse populations with body weights ranging from below 500 g to well over 70 kg, the reporting of pharmacological research is often limited by the lack of adequate stratifications according to age or developmental stage in CT design and analysis plans (6).

A clinical example of the pediatric PD endpoint knowledge gap is in evaluating the management of an oxygen-dependent newborn diagnosed with severe pulmonary hypertension (PH). For a pediatric study, the industry-standard, primary PD endpoints for PH trials include exercise capacity evaluated by the 6-min walk distance (8). This PD endpoint is clearly inappropriate for newborns and pre-ambulatory children. Pediatric PH PD endpoints need to account for the age and neurodevelopmental status of the child, and —in chronic conditions—take into account how these endpoints are expected to change over time as the disease progresses (9). Previously, pediatric PH studies have used various nonstandardized primary endpoints such as cardiac catheterization parameters and bicycle-ergometry evaluation of exercise tolerance (10). With variable PD endpoints used across studies, research cannot be compared or combined to determine which treatments are most effective in the pediatric PH population. Identifying validated, age-appropriate, condition-specific pediatric efficacy, and safety PD endpoints is a serious challenge currently faced by pediatricians, scientists, and drug developers alike.

This paper provides an overview of PD endpoints in children while exploring the scientific principles and challenges underlying the selection and validation of robust PD endpoints in children and neonates.

## Methods

Through the StaR Child Health network (2), an expert group was convened, including pediatric pharmacology and CT experts from the United Kingdom, Australia, and Canada. The StaR Child Health network is an international consortium working to improve global standards for child health research (2). A literature review guided by an experienced research librarian was performed to identify evidence about pediatric PD endpoint selection for clinical research. The evidence was summarized to provide the basis for recommendations for selecting pediatric PD endpoints and identify current knowledge gaps. MEDLINE (Ovid) and EMBASE (Ovid) were searched from 1946 to September 2017 to identify citations potentially relevant to the topic of “standards for assessing PD endpoints in children”. A search strategy incorporating the subjects of PD, practice guidelines, and “clinical trials as topic” with age-specific limits was developed (Supplement, online only). Guidelines from regulatory authorities (European Medicines Agency (EMA), FDA, and Therapeutic Goods Administration) were reviewed for existing standards relevant to establishing and measuring PD endpoints in children. Electronic searching was limited by a lack of standardized terminology and indexing of papers in the field. The broader electronic search identified papers specific to therapeutic areas, drug classes, and individual drugs, with no general standards for PD endpoints identified (Supplementary Table 1, online).

## Current regulatory guidance

To increase the efficiency of pediatric studies and maximize the use of existing information, regulatory authorities have accepted extrapolated data from trials in other populations (mainly adults). The FDA (US Food and Drug Administration) produced draft guidance in 2014 regarding general considerations for pediatric studies for drugs and biological products (11). It includes a pediatric study planning and extrapolation algorithm relevant to PD endpoints in children, reflecting an update of the FDA pediatric study decision tree previously published in 2003 (11). The decision tree addresses the circumstances under which full or partial extrapolation can be considered in children, given a similar disease progression and response to intervention. It is noteworthy that an FDA report highlighted that only 6% of drugs reviewed could be completely extrapolated from efficacy data in adults (12). If the efficacy or toxicity endpoint is delayed, rare, or cannot be directly measured, the FDA recommends the selection of a biomarker for this purpose (11). Available data on validated pediatric biomarkers are currently limited and, again, when extrapolating biomarker endpoints from other populations, age-dependent changes in the context of pediatric disease progression require consideration (7). The FDA has also started a Letter of Support Initiative where comments from the Center for Drug Evaluation and Research on the potential value of a biomarker can be appended (13). These letters are meant to increase transparency and provide contact information for other academic, industry, or government groups to provide collaborative data.

The FDA has published a table of biomarkers used as outcomes in the evaluation of FDA-approved therapeutics available online (14). Although this is undoubtedly helpful, little information is given on the context of use, and if pediatric patients were included in the evaluated studies. The Framework for Defining Evidentiary Criteria for Biomarker Qualification drafted by the NIH Biomarkers Consortium to harmonize the biomarker qualification process is available online (15), and further details regarding the qualification process are agency specific and outside the scope of this review.

The EMA Committee for Medicinal Products for Human Use (CHMP) has published several guidelines relating to drug development and PK in children (16) and in newborns (17). These guidelines are updated periodically, drafted by working parties, and subject to public consultation from relevant stakeholders. Disease-specific guidelines sometimes contain relevant PD standards in pediatric addenda (e.g., guidelines for pulmonary arterial hypertension (18), acute cardiac failure (19), and lipid disorders (20)). These addenda acknowledge the differences in the pathophysiology of these conditions between adult and pediatric patients and provide guidance to the pharmaceutical industry regarding trial design and appropriate endpoints in pediatric studies.

Regulatory–academic–industry partnerships have formed to accelerate the development of safe and effective medicines for children. An example of this partnership is the International Neonatal Consortium (INC) that includes nurses, clinicians, researchers, industry, regulators, and parent representatives. The goal of INC is to unite stakeholders, forge a predictable regulatory path, and to develop practical tools (21) that can be incorporated into clinical trials to increase efficiency (https://c-path.org/programs/inc/). The applicability of recommendations generated from such groups of international experts will likely extend beyond the neonatal period. Recently, INC released a white paper (22) to support investigators evaluating medicines in neonates that recommends linking PD data to PK where possible to determine the exposure–response relationships and interpreting this response in the context of available evidence in other populations. In October 2017, the FDA awarded a grant to the Institute for the Advancement of CTs for Children (I-ACT) and the Pediatric Trials Network (PTN) based out of Duke University in the United States (23). I-ACT and PTN share a goal of providing expert advice on CT design and conduct for drugs and medical devices in children. The Global Research in Pediatrics (GRiP) Network of Excellence, funded by the European Union has developed guidance and research tools for pediatric studies (24, 25, 26, 27, 28) that can be found in their publication repository (http://www.grip-network.org/index.php/cms/en/publications). The advantages of these international partnerships include harmonization, development of best research practice, and a global union of expertise and experience in pediatric clinical research.

## Selecting and measuring useful PD endpoints

Useful PD endpoints are defined as measures, which, when collected systematically, can inform decision making at the bedside, by policy makers and by health authorities (regulators). Several identified considerations that are relevant to selecting and measuring useful PD endpoints in children, infants, and neonates that include the use of biomarkers, modeling and extrapolation, compliance, outcome-scoring systems, and measurement validation are summarized below.

### Biomarkers as PD Endpoints

A biomarker is defined as a “characteristic that is objectively measured and evaluated as an indicator of normal biologic processes, pathogenic processes, or pharmacologic responses to a therapeutic intervention” (29, 30). Systematic reviews have identified a wide variety of biomarkers that have been used to measure response (31, 32, 33). Historically, many biomarkers validated for use in adults were inappropriately extrapolated to children without careful assessment of the influence of the developmental aspects of pathogenesis, ontogeny, and therapeutic response (7). There remains a dearth of validated biomarkers in pediatrics and neonatology, which has contributed to the “PD knowledge gap” recognized in pediatric drug development (34, 35). Validated biomarkers facilitate prediction of disease progression and treatment response. Importantly, they can provide useful surrogate endpoints within the trial design, when they have reliably demonstrated that they can predict the relevant clinical outcomes (35). In order for biomarkers to serve as valid pediatric PD endpoints, changes in biomarker concentrations must also predict beneficial or harmful clinical response in the population of interest (22).

The use of biomarkers in clinical trials is highly context specific, and regulators require that high standards of validation be met to accept biomarker data during the marketing authorization process. For example, the FDA (36) and EMA (37, 38) launched formal biomarker qualification processes and provide scientific advice to support innovation in this field. In an effort to disseminate information regarding the established biomarkers, the FDA established the BEST (Biomarkers, EndpointS, and other Tools) (36) resource that contains examples and explanations. During the FDA’s review of new molecular entities, several studies reported the use of biomarkers as outcomes in clinical trials (39). For example, biomarkers used to evaluate inborn errors of metabolism included blood cell count, growth, serum low-density lipoprotein-C, blood phenylalanine, forced vital capacity, hemoglobin, plasma concentrations of ammonia, glutamine, and citrulline, as well as splenic volume measured by MRI (39).

In situations where a biomarker is known to change with age as a child develops, an *a priori* correction may be applied to account for age. One such method is to convert biomarker measures into a *z*-score. A *z*-score follows the standard normal distribution (i.e., mean of 0 and standard deviation of 1). Hence, the *z*-score gives the magnitude and direction of deviation from the expected value for age in standard-deviation units, with a *z*-score of zero representing a child having the expected biomarker value for their age. An example of *z*-scoring in PD modeling is the measurement of CD4 T-lymphocyte count in HIV-infected patients treated with antiretrovirals. The adaptive immune system develops rapidly during early childhood, and while the thymus may cease growing after 1 year, changes in the epithelial space occur over the first two decades of life. The implication of this is that the normal CD4 T-cell count drops threefold during childhood, stabilizing in early adolescence (40). CD4 T-cell reconstitution following antiretroviral initiation has been modeled using *z*-scores (41). One potential criticism of *z*-score conversion is the strong underlying assumption that the study population has the same age-dependent distribution values as an available reference population from which the *z*-score is calculated (42). Where appropriate, juvenile animal models may shed light on the relationship between growth and development with variability in PD endpoints.

When evaluating biomarkers in pediatric drug development, clinical, regulatory, and methodology experts should be consulted as early as possible in the study-design process. Biomarkers must be developed and tested within a specific context of use in drug development that includes the class of biomarkers and the specific research question (33). The financial cost of validating biomarkers to meet regulatory standards and the time frames involved must not be underestimated. This is an area where dedicated funding streams will be critical to enable sustained progress in biomarker development and to underpin future progress in pediatric therapeutics.

### Modeling: Extrapolating Exposure–Response Relationships

Several challenges arise when extrapolating exposure–response (PK–PD) relationships across indications and age-groups. One fundamental source of uncertainty is a lack of reliable data on disease progression, particularly in neonates and young children who were historically excluded from clinical research. Without clear knowledge of the differences in disease progression, it is problematic to use one population to predict clinical response in another (43). Although it is increasingly possible to make assumptions about the impact of age-related changes in enzymes, transporters, and organ function on a drug’s PK, it is often less clear how these variables affect PD response (44). A further challenge occurs when several medications with multiple mechanisms of action are coadministered, such as combined chemotherapy in oncology.

Modeling and simulation are increasingly required by regulators to inform the design and conduct of PK/PD studies for drugs used in pediatric populations (11, 45). A recent report by Mulugeta *et al.*, identified 31 products approved in children by the FDA between 1998 and 2012 with full (39%) or partial (61%) extrapolation (43). Most of these products (*n*=25/31) were studied in more than one other pediatric age group (37). PD can be modeled alone, or simultaneously with PK (25, 46, 47). A detailed overview of the principles of PKPD modeling is available elsewhere (47, 48, 49, 50). In brief, a PK model describes the relationship between the drug-dosing regimen and the plasma drug concentration time profile, whereas a PD model describes the relationship between drug concentration and the biological effect (48). Mathematical techniques for creating models of the interactions between developmental physiology, PK, PD, and disease (pharmacometrics) have become increasingly sophisticated in recent years, resulting in the successful application of PD models in pediatric drug development and post-marketing research (47, 51, 52, 53, 54).

Furthermore, PKPD models can be used to inform the design of comparative randomized control trials (45, 53, 55). In some cases, simulation and extrapolation from robust adult PKPD data are sufficient for licensing medicines in children, for example, esomeprazole for gastroesophageal reflux disease in children aged 1–17 years (56). PKPD models can play an important role in determining if the expected outcome effect size will be similar in children of different ages and make the best use of available data in other populations (57). For example, the new class of antidiabetic medications sodium-glucose cotransporter 2 (SGLT2) inhibitors exert their effects by promoting renal glucose elimination, through reduction of glucose reabsorption in the proximal tubule (58). Adolescent patients with type-2 diabetes tend to have superior renal function to adult patients, and hence SGLT2 inhibitors will potentially be more effective at equivalent doses. Quantifying this with modeling can thereby reduce CT sample size requirements using a predicted increased effect size in the adolescent age-group.

### Compliance and PD Response

Patient adherence with the therapeutic intervention can influence the evaluation of treatment differences between study arms. Sophisticated research into pediatric PD in explanatory trials needs to be accompanied by consideration of medication adherence, particularly in ambulatory settings and in complex diseases. Although adherence is often considered separately from PD endpoints of treatment success, the consequences of disregarding patient adherence (or compliance) during the early phases of drug development are wide-reaching as nonadherence to treatment regimens can reduce or prevent the detection of treatment efficacy, thus having an impact (potentially significantly) upon trial results (59). Achieving medication adherence can be highly challenging in the pediatric setting (60). Various innovative ways of evaluating adherence in pediatric clinical studies, including electronic measurement (61), and measurements of drug and metabolites through various media (62, 63) merit further research.

### Outcome-Scoring Systems

Scoring systems, which assign numerical merit to outcomes to estimate the degree of a clinical situation, remain popular in pediatric medicine (53, 64). Scoring systems can support pediatric PD measurement in CTs by combining several isolated measures into one score to monitor disease status before and after therapeutic intervention(s). However, scoring systems derived for adults again may not be suitable or valid for use in the pediatric population (65). Dedicated age-specific scoring systems need to be developed and then validated for use as endpoints in CT settings, as well as within clinical practice (65, 66, 67). Examples of age-appropriate PD-related scoring systems include the COMFORT-B score (68) used to measure sedation in pediatric critical care and the Premature Infant Pain Profile (69) used to measure pain in nonverbal children. In adults, the gold standard for measuring pain is self-report, which is clearly inappropriate for younger age groups. The COMFORT-B score is made up of six items: alertness, calmness, respiratory response (for ventilated children) or crying (for spontaneously breathing children), body movements, facial tension, and muscle tone. Each item is rated on a scale 1–5 so that the total score ranges from 6 to 30. The COMFORT-B score has been validated in this clinical setting, and if CT use of COMFORT-B is planned, outcome assessors should be trained with video assessment and test patients (68). Satisfactory results compared with an experienced scorer to measure inter-rater reliability (70, 71) should be achieved before collecting PD CT data. However, while scoring systems confer the advantage of combining multivariate data into a univariate quantity (72), they sacrifice the granularity of detail within the data. Ideally, it is preferable to retain all raw data used to generate composite scores whenever feasible, to facilitate more detailed statistical analysis. For example, the Finnegan Score (73) for neonatal abstinence syndrome includes 21 signs and symptoms. If raw data are maintained, investigators may be able to answer more specific research questions regarding the intervention’s effect on generalized convulsions (one of the score items).

### Validation of PD Endpoints

Although the validation process presents practical and financial challenges, it is essential to ensure that PD endpoints are meaningful, reproducible, and relevant (3). There are several examples of validated pediatric PD endpoints (5). The aforementioned COMFORT-B score presents a useful example. Given the potential limitations of a scoring system vulnerable to interobserver variability, Ista *et al.* (74) ensured reliability by training PICU nurses with video material and bedside instructions. Nurses new to the scoring system underwent repeated assessments with a trained nurse to maximize the fidelity of their scoring. Such a rigorous approach to staff training benefits the application of scoring system-based PD endpoints in both clinical and research settings, and is strongly recommended for all PD endpoints that depend on proxy (healthcare professionals’ or parents’) observations or measurements.

In the analytical setting, the validation of laboratory biomarker assays can present additional difficulties. This was demonstrated in the management of type-1 diabetes, where long-term monitoring of HbA_1c_ (glycated hemoglobin) was affected by technical aspects of the measurement methods. This led to speculation over whether the differences in patients’ longitudinal HbA_1c_ measurements were due to the laboratory assays rather than differences in clinical management strategies: details of the HbA_1c_ assay laboratory and equipment factors are discussed elsewhere (75, 76, 77, 78). These experiences highlight the importance of the new regulatory guidelines on bioanalytical method validation (79, 80), as well as inter-laboratory proficiency testing and methodological standardization for both old and new biomarker assays (78). Quantitative biomarker assays should be reliable, selective, and validated under conditions equivalent to good laboratory practice whenever feasible (3). For multicenter-CTs, transference studies should be planned prospectively to quantify variability related to laboratory instrumentation.

Clinical aspects of validation include the definition of the normal ranges/values of PD markers (including biomarkers) in both healthy children and the target patient population. The latter can be difficult, given the rarity of many pediatric diseases, which affect small, heterogeneous patient populations (81). The identification of age-matched controls can be challenging. Developing suitable pediatric reference value distributions thus requires dedicated research in its own right (82). There is ongoing research into the identification and validation of pediatric biomarkers in different subspecialist clinical contexts (32, 83, 84, 85). For example, there have been rapid advances in candidate biomarkers for renal function and acute kidney injury in children, such as cystatin C, neutrophil gelatinase-associated lipocalin, and kidney injury molecule 1 (86, 87, 88), although translation from research into clinical practice has been slow (89). The use and uptake of validated biomarkers will be improved by efforts of regulators to swiftly disseminate new information and updates regarding biomarkers relevant to both clinical practice and CT outcomes in children.

## Identifying a useful PD endpoint

PD endpoints must be able to consistently quantify the clinical response of a child to a specific intervention, as measured at a specific stage of development. Valid PD endpoints are described within the context of the “four Rs”: relevant, responsive, reproducible, and reliable (90, 91). The characteristics of an ideal PD endpoint are summarized in Table 1. PD endpoints must be clinically relevant and, where possible, should take into account how the patient feels and functions (92). PD endpoints must be responsive to changes in clinical progression over time; this characteristic is essential to track disease improvements and treatment progression (92).

To ensure validity, PD endpoints should be reproducible, for example, in patients of different ethnicities. Due to age-dependent variability in rates of disease progression and organ function in children, different PD endpoints in specific age groups (e.g., neonates, toddlers, and adolescents) are typically required. Within these age groups, PD endpoints must be reliable and should be similar, regardless of the environment or the person who takes the measurement. To determine which PD endpoints should be evaluated, we recommend undertaking a systematic review and using a consensus process to select outcomes and measurement tools. Patients and families, as well as policy makers should be included in the process to select meaningful PD endpoints. Methods for the development of disease-specific core outcome sets are described by Williamson *et al.* (93). A repository of core outcome sets is available online through the Core Outcome Measures in Effectiveness Trials (COMET) initiative (http://www.comet-initiative.org/). If a core outcome set exists for the condition of interest, it should be employed, whereas additional relevant PD endpoints may be added.

## Current PD research needs

Stemming from a review of the currently available regulatory and scientific evidence, recommendations for future research for specific disease areas are presented in Table 2. In order to increase the quality, consistency, and usefulness of PD endpoints, targeted methodology research is needed. For example, as outlined above, scoring systems comprising a composite of several measures have a risk of losing information since different combinations of subscores can give the same overall score. Item response theory approaches could be useful in teasing out contributions of the individual subscores to the PD response (72). The heterogeneity of definitions of PD endpoints exacerbates the difficulty in introducing generic recommendations; however, efforts to harmonize approaches internationally will begin to address these issues.

A further challenge for synthesizing scientific literature and guidance in this area includes a lack of appropriate and valid search terms. Ideally, the development of MeSH search terms would improve the ability of future researchers to identify, compare, and contrast pediatric PD marker validation studies. As the validation of PD endpoints often requires large sample sizes, there is an important role for industry–academic–regulatory partnerships to moving the science behind selecting valid PD endpoints forward. Data from previous studies where PK–PD may not be the primary objective, could potentially be used by researchers to better characterize the PK–PD relationships in pediatric diseases where such information is lacking. This will require open data-sharing agreements and partnerships. Additional research is needed into the ability of real-world evidence (routine laboratory tests, patient registries, and electronic health records) to support the selection of high-quality pragmatic pediatric PD endpoints. Recently, the use of patient-reported outcomes in clinical practice has increased; however, the validation of patient-reported outcomes to meet regulatory requirements should be explored. Finally, how to best engage patients and families in the process of selecting PD endpoints requires further evaluation.

## Conclusions

The importance of utilizing appropriate pediatric PD endpoints, including biomarkers, and facilitating their validation in children is clear. Although there is increasing knowledge of PK in neonates and children, there is a paucity of information related to PD. Based on the available evidence, no overall recommendation for the selection of pediatric PD endpoints in CTs can be provided at this stage. However, we provide criteria for selecting and measuring useful PD endpoints for each age-disease-specific group, and set an agenda for research in this field. Engaging with regulators early in the development process will help ensure that endpoints meet the regulatory validation requirements. Trial sponsors and regulators need to agree to use early-phase trials to validate candidate PD endpoints, including biomarkers, to advance the field. Across these domains, PK/PD modeling enhances both pediatric PD research and trial design, and, where resources permit, model-based approaches should underpin future pediatric PD research.

## Figures and Tables

**Table 1:** Seven characteristics of useful PD endpoints

1. Meaningfully describe the patient’s pharmacological and clinical responses to drug therapy with respect to
(a) Incorporating both harms and benefits
(b) Accounting for patient and families well-being (quality of life)
2. Can be interpreted against data extrapolated from other diseases or age-groups and existing scientific literature
3. Can be used to answer the research question while informing healthcare decision making at the bedside and policy level
4. Is responsive to change and comes with a defined age-specific minimally important difference
5. Is reproducible and, where possible, objective
6. Can be consistently and reliably measured by outcome assessors
7. Has an established age-appropriate validated measure with established reference ranges in the specific age group and disease state
8. Is feasible with respect to
(a) Acceptability in terms of burden on the child or caregivers with minimal compliance/adherence concerns
(b) Timing: where possible combined with routine tests
(c) Cost considerate: license, equipment, and skill set of the outcome assessor

**Table 2:** Six disease-specific research activities needed to advance pediatric pharmacodynamics

1. Develop, test, and implement validation strategies for pharmacodynamic measurement tools (including biomarkers), especially in younger age-groups
2. Harmonize the definitions of disease and disease severity (if not already agreed)
3. Develop methods (akin to allometric scaling in pharmacokinetics) for robust scaling of pharmacodynamic endpoints with known age- or size-related factors
4. Identify the optimal pharmacodynamic study design and sampling times
5. Develop and test the study design methods for determining what outcomes are important to patients, families, and policy makers
6. Determine baseline “normal” values for biomarkers accounting for developmental status and disease progression
7. Develop, test, and implement MeSH terms for indexing of papers, developing, and validating pharmacodynamic measures in child health trials to ensure that future literature searches will identify these articles
